# Supplementary material for: Patterns of Intron Gain and Loss in Fungi
Source: PLoS Biol. 2004 Nov 30;2(12):e422. doi: 10.1371/journal.pbio.0020422 (PMC532390; doi:10.1371/journal.pbio.0020422)
Supplement: Table S1 — Also available at http://genes.mit.edu/NielsenEtAl/. (4.3 MB ZIP). [file pbio.0020422.st001.zip › NielsenEtAl/html/1357.html]

AN6453.1.NCU02196.1.MG06615.1.FG00162.1


```
 CLUSTAL W (1.82) Multiple Sequence Alignments - Introns Inserted


Sequence 1: MG06615.1	419 aa
Sequence 2: FG00162.1	422 aa
Sequence 3: AN6453.1	377 aa
Sequence 4: NCU02196.1	425 aa
Alignment Length: 451 aa
Number Identitical Residues: 161 aa
Alignment Score (without introns) 9014


MG06615.1 	MAAAAVLTPAA------------------QPDIQYAPDIEKWHQRTAKRFREENDRLQKT
NCU02196.1	MATAAVQVSVP--------------APVGQPDIGYAPDHDKYLARVKRRRENEK--LESS
FG00162.1 	MATAAILTPVAPPSAPTFGTTPAAQPPPGQPDISYHPNWETYQARVARRTQTEN--LSKE
AN6453.1  	MSVVIPPVPV---------------GPDGQPDIQYLPDPVKYAARAERRQREEN--LPRT
          	*:..   ...                . .**** * *:  .:  *. :* . *:  *   

MG06615.1 	LPEGFPAQLQGSLVWEGKTVGQSYDWTYVLNEEQLAEIDDAVRHFKS1LNLPLGHIDAAT
NCU02196.1	LPPGFPRRLDSDLVWDGNTLAETYDWTYRLTEEAIDEIEAALRHFKS1LNKPLGYINQET
FG00162.1 	IPEGFPKELKGDLVWEGETVAKEYKWTYTFTDEQLEDINNALAHFKS1LNIAKGYISQET
AN6453.1  	LPEGFPQELKSDLAWDGKTVGEVYDWNYHLTEDDLKEVDQALLHFKT1LNLPIGLVNQET
          	:* *** .*...*.*:*:*:.: *.*.* :.:: : ::: *: ***: ** . * :.  *

MG06615.1 	FPLPKTRPELRRLSRELHDGHGFFVIRGVDVDR~HTREENIIIYAGVSSHIAAVRGRQDS
NCU02196.1	FPLPRLHHTLRSLSHELHHGHGFKVLRGLPVTS~HTREENIIIYAGVSSHVAPIRGRQDN
FG00162.1 	FPLPKTHDELRRLSRELHFGHGFFVLRGLDVDR~YTREENIIIYAGISSHIGSIRGRQDA
AN6453.1  	FPLYKLHNSLRLISREIHLGRGFKVIRGVPVDH2P---------------------RPPG
          	*** : :  ** :*:*:* *:** *:**: *                         *   

MG06615.1 	TFNGTKADVVLSHIKDLTLQLQQQQEARAAKISIGSPAYTADKQVFHTDSGDIVSLFALG
NCU02196.1	QHNGHPADVVLAHIKDLS-------TTVSDVSKIGAPAYTTEKQVFHTDAGDIVALFCLG
FG00162.1 	YYDGKRADVVLGHVKDVR---------STTKIKIGSPAYTADKQVFHTDSGDIVSLFALG
AN6453.1  	QQLAGKADVVLAHITDLS-------RKV-DTKTIKAPAYTTEKQVFHTDAGDVITLFALQ
          	   .  *****.*:.*:               .* :****::*******:**:::**.* 

MG06615.1 	EAAEGGASKLASTWRVYNELARTRPDLIHTLAADWPLEN2FDKST--SERYFSRPLVYHQ
NCU02196.1	EAAEGGQSYLSSSWKVYNELAATRPDLVRTLAEPWVADE~FGKEG---RKFSVRPLLHFQ
FG00162.1 	TAAEGGASRLASAWRVYNHIAETRPDLIRTLAEDWEAEL2FTKTVNDTDKVSRRPLIHHQ
AN6453.1  	EAAEGGQSYLSSSWHVYNELARTRPDLIHTLAEPWVAEQ~GGD-------VVSRPLLYYQ
          	 ***** * *:*:*:***.:* *****::***  *  :    .          ***::.*

MG06615.1 	PASE--KQP-----ERILLQYARRYLVGFGALPRSADVPPITEAQAEALDALHFLGERFH
NCU02196.1	STAAAASREAKPESERLIIQYARRTFTGYWGLPRSADIPPITEAQAEALDALHFTAEKYA
FG00162.1 	PASTDGKTP-----ERVVLQYARRYYVGFGALPRGNHIPPITEAQAEALDTLHFLGERFS
AN6453.1  	PQTSSSP-------ERLIIQYARRGFTGYWGKPRAATLPPITEAQAEALDALHYLAEKSA
          	. :  .        **:::*****  .*: . **.  :************:**: .*:  

MG06615.1 	VATDFRKGDMQYVNNLAVFHARDGFRDTVDKQ2RHLVRLWLRDPENAWQTPERLRTRWSR
NCU02196.1	VALDFRQGDVQFVNNLSVFHSRAGFRDEGEKQ~RHLVRLWLRDPENAWETPEALKERWER
FG00162.1 	VSTDFQKGDIQYVNNVAVFHARDGFRDTEDQQ2RHLLRLWLRDPEYAWETPAALSERWER
AN6453.1  	VALDFHKGDIQVINNLSIFHARAGFTDSDEKR~RHLVRLWLRDPELAWETPERLKSRWEY
          	*: **::**:* :**:::**:* ** *  ::: ***:******** **:**  *  **. 

MG06615.1 	LYDGVTPESQVLPLEPFVRGASNGAD~RVNK-----
NCU02196.1	VYGGVSPEREVFPLEPQIRSASKGES~VGTQGGGGY
FG00162.1 	VYGGVTPELQTFPLEPYVR------I1RF-------
AN6453.1  	VYDGVDAAKSIFPLKPFVRSQ-----~---------
          	:*.** .  . :**:* :*.
```
